# Data showing phenotypic profile of uropathogenic *Escherichia coli* isolates from sepsis patients

**DOI:** 10.1016/j.dib.2016.03.047

**Published:** 2016-03-17

**Authors:** Vivek Verma, D. Nagarjuna, Gajanand Mittal, Parveen Kumar, Rakesh Singh Dhanda, Rajni Gaind, Manisha Yadav

**Affiliations:** a Dr. B.R. Ambedkar Center for Biomedical Research (ACBR), University of Delhi, Delhi 110007, India; b Department of Microbiology, Vardhman Mahavir Medical College (VMMC) and Safdarjung Hospital, Delhi 110029, India; c Department of Translational and Regenerative Medicine, Post Graduate Institute of Medical Education & Research (PGIMER), Chandigarh 160012, India

**Keywords:** *Escherichia coli*, Phenotypes, Sepsis

## Abstract

Bacterial virulence factors (VFs) influence the site and severity of urinary tract infections (UTI) and further leading to sepsis infection. Phenotypic characterisation of VFs specific to sepsis *Escherichia coli* strains has not been characterized in Indian population till date. In this data article, we have described important VFs of uropathogenic *E. coli* (UPEC) that is P fim, Type-1 fim, cell surface hydrophobicity, mannose resistant haemagglutination/mannose sensitive haemagglutination (MRHA/MSHA) expression and α-haemolysin production. The data includes a profile of the five VFs investigated in *E. coli* isolates from sepsis patients (*N*=78) and control group (*N*=50) from non-sepsis subjects. We found that P fim phenotype was expressed in 25.3% of *E. coli* isolates from sepsis patients, whereas Type-1 fimbriae was detected in 30.5%. Cell surface hydrophobicity phenotype was present in 30.5%, α-haemolysin in 26.3% and MRHA/MSHA in 22.1% of sepsis *E. coli* isolates. None of the control *E. coli* isolates showed presence of these phenotypes. The combined phenotypic profile of all the five VFs was significantly higher in sepsis patients as compared to the control group.

## **Specifications table**

**Table:** 

Subject area	*Biology*
More specific subject area	*Medical microbiology, E. coli*
Type of data	*Graphs*
How data was acquired	*Phenotypic assay of E. coli strains*
Data format	*Analyzed*
Experimental factors	*Confirmed sepsis patients*
Experimental features	*Phenotypic profile of E. coli isolates from Sepsis patients*
Data source location	*New Delhi, India*
Data accessibility	*Data is with this article only*

## **Value of the data**

• First report of phenotypic profile of the uropathogenic *Escherichia coli* isolates from sepsis patients in the Indian population.
• The data showed that P fim and Type-1 fimbriae phenotype were highly expressed in *E. coli* isolates from sepsis patients, indicating their important role in adherence.
• A high expression of α-haemolysin in the *E. coli* isolates is indicative of induction of toxicity.
• Information of the phenotypic profile of the sepsis patients in response to *E. coli* infection can be helpful in understanding the role of VFs in adherence to host epithelial cells and induction of toxicity among such patients.

## 1. Data

**Fig. 1:**
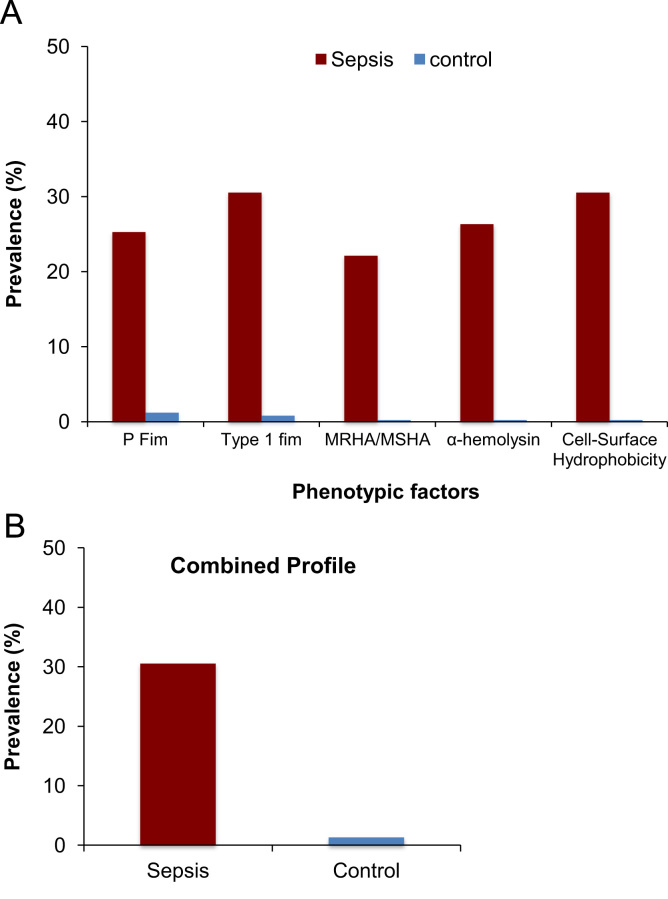
(A) The phenotype profile of five important virulence factors of *E. coli* in sepsis patients and control group. (B) Combined virulence profile of sepsis *E. coli* samples as compared to control group.

The phenotypic profiling of important virulence factors (VFs) have shown that P fim phenotype was expressed in 25.26% of *E. coli* isolates of the sepsis patients, whereas Type-1 fimbriae was expressed in 30.52% of *E. coli* isolates by haemagglutination (Fig. 1A). The expression of P fim and Type 1 fimbriae was significantly higher in sepsis *E. coli* isolates as compared to control group (*p*<0.01). Cell surface hydrophobicity phenotype was present in 30.52% of *E. coli* isolates whereas 26.31% were expressing α-haemolysin and MRHA/MSHA phenotype was shown by 22.1% of *E. coli* sepsis isolates (Fig. 1A). Similarly, the cell surface hydrophobicity, haemolysin and mannose resistant phenotypes were significantly higher among sepsis *E. coli* isolates as compared to the control group (*p*<0.01). Further combined expression profile of five phenotype virulence factors was significantly higher in sepsis *E. coli* isolates as compared to control group (*p*<0.001) (Fig. 1B).

## 2 Experimental design, materials, and methods

### 2.1 Collection and culturing of clinical *E. coli* isolates

*E. coli* strains (*N*=128; Sepsis=78; Control=50) were obtained from the stock library of Department of Microbiology, Vardhman Mahavir Medical College and Safdarjung hospital, New Delhi, India. The *E. coli* strains were collected from confirmed sepsis patients who visited the hospital while control group consists of the faecal *E. coli* isolates from non-sepsis controls. The bacteria were grown on tryptic soy agar (TSA) agar plates at 37 °C overnight and further stored at 4 °C for the phenotypic characterisation.

### 2.2 Haemagglutination assay: P-fimbrial/Type 1 fimbrial phenotype

The phenotype of P-fimbrial was defined by P blood group dependent haemagglutination [1], [2]. P-fimbrial expression was defined by agglutination of P1 (receptor positive) but not p (receptor negative) erythrocytes. Type 1 fimbrial was detected by haemagglutination of human and guinea pig erythrocytes after in vitro passage in Luria broth. Agglutination was performed in the presence and absence of α-methyl-D-mannoside. Strains causing mannose-sensitive agglutination were defined as Type 1 fimbriated [3].

### 2.3 MRHA/MSHA assay

Haemagglutination was performed in round-bottomed microtitration plates. One drop (100 µl) of bacterial suspension was mixed with one drop of erythrocytes (human A^+ve^, 3% v/v in 1× PBS) and one drop of PBS, with or without D-mannose (3% w/v). The plate was left to rotate (15 rpm) for 5 min at 25 °C followed by rotation for 5 min at 4 °C. Haemagglutination was considered to be mannose-resistant (MRHA) when it occurred in the presence of mannose and mannose-sensitive (MSHA) when it was inhibited by mannose [4].

### 2.4 Cell-surface hydrophobicity

The cell-surface hydrophobicity was calculated by the salt aggregation test (SAT) with suspensions (5×10^9^ cfu/ml) in 0.2 M phosphate buffer, pH 6.8, of bacteria grown on TSA medium. In brief, suspensions were mixed with ammonium sulphate solutions at final molar (*M*) concentrations of 2.0, 1.4, 1.0, 0.4, 0.1, 0.06 and 0.02. Strains were considered to be hydrophobic when they aggregated in ammonium sulphate at concentrations ≤1.4 M [5].

### 2.5 α-Haemolysin production

Sheep blood agar plates were used for determination of α-haemolysin production that contained 1% sheep blood (v/v). About 7–8 wells of 8 mm diameter were made on blood agar plate and 50 µl of bacterial lysate was poured into wells and incubated overnight. Zone of inhibition was recorded. Strains with a clear halo after overnight culture at 37 °C were defined as haemolytic [6].

## 3 Statistical analysis

The chi-square test was used for statistical comparison between the two groups. *P* values≤0.05 were considered as statistically significant.
